# Gut-Brain-axis: effect of basil oil on the gut microbiota and its contribution to the anticonvulsant properties

**DOI:** 10.1186/s12906-023-04211-5

**Published:** 2023-11-03

**Authors:** Sumaiya Bandile Amidu, Vivian Etsiapa Boamah, Edmund Ekuadzi, Priscilla Kolibea Mante

**Affiliations:** 1 Department of Pharmacology, Kwame Nkrumah University of Science and Technology, Private Mail Bag, Kumasi, Ghana; 2 Department of Pharmaceutics, Kwame Nkrumah University of Science and Technology, Private Mail Bag, Kumasi, Ghana; 3 Department of Pharmacognosy, Kwame Nkrumah University of Science and Technology, Private Mail Bag, Kumasi, Ghana

**Keywords:** Acetate, Antibiotics, Bacteroidetes, Diazepam, Epilepsy, Firmicutes

## Abstract

**Background:**

Epilepsy is a chronic neurological condition that disrupts the normal functioning of the brain and it is characterized by seizures. Research suggests the involvement of the Gut-Brain axis in epilepsy. This study seeks to determine the role of the gut microbiota in the anticonvulsant effect of basil oil (BO) using antibiotic-depleted and altered germ-free mice against naïve mice in Pentylenetetrazole (PTZ) induced seizure model. There is an ever growing interest in improvement of treatment outcomes in epilepsy and also in the development of newer therapeutic options, especially in the population of patients that do not attain seizure relief from available antiseizure medications (ASMs). According to research, gut microbiota can alter brain function and development. Increasing evidence suggests disrupting the delicate symbiotic balance existing between the gut and brain results in disease conditions. Also, the oil from *Ocimum basilicum L.*, (BO) has been proven scientifically to significantly block clonic seizures induced by PTZ and picrotoxin in seizure models.

**Methods:**

The microbiota of mice were depleted or altered by administering cocktail antibiotics and individual antibiotics respectively. DNA was isolated from mouse stool, and then the 16S ribosomal ribonucleic acid (16S rRNA) gene was quantitatively amplified using reverse transcription-polymerase chain reaction (RT-PCR). Amplicons were sequenced to determine the phylogenetic make-up of the bacteria involved. Metabolic profiles of the serum and stool of mice were determined using Proton (1H) Nuclear Magnetic Resonance (NMR) spectroscopy.

**Results:**

Cocktail antibiotic pre-treatment significantly reversed the anticonvulsant effect of BO by increasing frequency and duration of seizures but did not affect latency to seizure. In mice pre-treated with single antibiotics, the anticonvulsant effect of BO was lost as latency to seizures, frequency and duration of seizures increased compared to mice that received only BO. Assessment of the phylogenetic make-up of the microbiota in antibiotic pre-treated mice showed a distorted composition of the microbiota compared to the control group.

**Conclusion:**

Depletion of the microbiota significantly reversed the anticonvulsant actions of BO. The concentrations of short chain fatty acids (SCFAs) was higher in stool than in the serum of the mice. Administration of BO probably does not influence the microbial composition within the mouse microbiota. The elevated ratio of Firmicutes to Bacteroidetes in microbiota-depleted groups might have contributed to the reversal of anticonvulsant actions of BO.

## Background

Epilepsy is a chronic neurological disorder characterized primarily by recurrent and unpredictable disruptions in normal brain function, which are typically caused by uncontrollable neural excitation in the brain [17, 45, 51]. The physical manifestation of epilepsy is a seizure [37]. An epileptic seizure is a brief period of signs and/or symptoms caused by abnormally high or synchronous neuronal activity in the brain [17]. The underlying pathophysiology behind epilepsy disease progression remains unclear. Antiseizure medications (ASMs) act to reduce the frequency of seizures symptomatically [34]. Alternative approaches to managing epilepsy is on the rise as approximately 30% percent of patients with epilepsy do not get relief from any ASMs currently available [4, 45, 49, 51]. It is agreed that the brain communicates with the gastrointestinal tract via multiple pathways [38]. The communication between these two exists bidirectionally and has been dubbed the gut-brain axis [5, 6]. This axis is composed of a wide range of systems including the central nervous system (CNS), the autonomic nervous system, enteric nervous system, hypothalamic–pituitary–adrenal axis, neurotransmitters, hormone and neuropeptides, intestinal microenvironment, enteroendocrine, immune system, and the blood–brain barrier [61]. These systems work hand in hand to ensure the efficient functioning of the gut-brain axis. In the gut-brain axis, the neural or nervous, endocrine, immune and the enteric nervous systems (metabolic) are the key pathways through which this communication occurs. The gastrointestinal tract (GIT) provides the platform for these pathways [11, 12, 33]. The aforementioned pathways are strongly impacted by the gut inhabitants, specifically the microbes [5]. The influence of this communication has been recognized in instances where the microbiota induce cells in the gastrointestinal tract to synthesise neurotransmitters or neuro active molecules such as gamma aminobutyric acid (GABA), catecholamine, acetylcholine, histamine, melatonin, 5-hydroxytryptamine (5HT) or serotonin and SCFAs (that possess neuroactive properties) that modify the function of brain [5, 11, 44].

In relation to epilepsy, the microbiota in murine models play an important role in guiding brain development and neurological behaviour. The gut microbiota influences carbohydrate and amino acid metabolism, microglial and astrocytic function, vagal neuronal activity, and hippocampal neurotransmitter levels, all of which are important in epilepsy [35]. Also, basil has been used for CNS disorders and seizures with studies indicating that the essential oil of basil may exert its pharmacological properties through interactions with the GABAergic system and voltage-gated Na^+^ channels thereby reducing seizure activity [58]. Scientists have reported that *Ocimum basilicum L.* increased the latency time of the onset of clonic seizures in a dose-dependent manner. Hence, *Ocimum basilicum L* decreased the percentage of animals showing convulsion in response to intraperitoneal (i.p.) injection of pentylenetetrazole (PTZ) [58]. In another study, *Ocimum sanctum*, another species of the genus *Ocimum* in combination with levetiracetam, in PTZ-kindled rats decreased seizure scores. This combination showed maximum protection against PTZ-induced seizure, which indicates that *Ocimum* per se has anticonvulsant potential, and in combination with levetiracetam has additive effects [47]. Furthermore, a study indicated that intraperitoneal injection of *Ocimum basilicum* hydroalcoholic extract was effective against epileptic parameters in mice and confirmed the antiepileptic property of the leaves of this plant.

This study sought to determine the role of the gut microbiota in the anticonvulsant effect of basil oil using antibiotic-depleted and altered mice against naïve mice in the PTZ-induced seizure model. This was achieved by confirming the anticonvulsant activity of BO using the PTZ test, depleting and altering the gut microbiota by treating mice with cocktail and individual antibiotics respectively. The metabolite profiles of serum and stool samples in naïve and germ-free mice were determined by Proton (1H) Nuclear Magnetic Resonance (NMR) spectroscopy-based metabolomics. The difference in anticonvulsant activity of BO in naïve and germ-free mice was determined by a PTZ-induced seizure test. The phylogenetic composition of bacteria within the intestinal microbiota of both naïve and germ-free mice was determined through the utilization of quantitative reverse transcription-polymerase chain reaction (RT-PCR) in conjunction with 16S ribosomal RNA (rRNA) gene sequencing.

## Materials and methods

### Materials

#### Drugs and chemicals

Basil oil was purchased from Sigma-Aldrich USA; Quick-DNA™ Fungal/Bacterial Miniprep Kit (Zymo Research); Primers were from Inqaba biotec (United Kingdom). Pentylenetetrazole (PTZ), Neomycin (neo), 3-(Trimethylsilyl)-1- propanesulfonic acid-d6 sodium salt (DSS-d6), Deuterium oxide (D_2_O) and Sodium Azide (NaN_3_) were acquired from Sigma-Aldrich USA, while Diazepam (DIA), Vancomycin (van), Ampicillin (amp), Metronidazole (met), Amphotericin B (ampho-B) and normal saline were purchased locally from Lansah Chemist, Ghana for the study. All drugs were dissolved in normal saline while basil oil was suspended in small amounts of tween 80 and normal saline.

#### Experimental mice

Institute of Cancer Research (ICR) male mice (25–35 g; 6 weeks old) were used in this study. A total of 98 mice were used in the study. Mice were purchased from Centre for Plant Medicine Research (CPMR) in the Eastern region of Ghana. The mice were allowed to acclimatize to the environment for seven days prior to any experimental procedure. Mice were housed in groups in stainless steel cages at the *vivarium* of the Department of Pharmacology, Kwame Nkrumah University of Science and Technology (KNUST). Mice were fed with normal rodent diet. They were maintained in a 12-h day and night cycle where food and water were provided ad libitum*.* All experimental protocols used in this study adhere to the standards established by the KNUST Animal Ethics Committee (KNUST 0012) and the Institute for Laboratory Animal Research's Guidelines for the Tutelage and Handling of Experimental Organisms [20].

##### Experimental Samples

Prior to extraction or any other process, all mouse faeces utilized in this study were randomly collected from metabolic cages in the morning, immediately frozen at -20 °C, and stored for a period of 2 to 6 weeks. Therefore, it is impossible to link these stools to a single animal.

### Instrumentation

HORIBA Medical: Pentra C200 auto analyser was used in the analysis of biochemical parameters. Blood samples were analysed for haematological parameters using HORIBA Medical: Yumizen H500 analyser. Tubes containing blood samples were centrifuged with Thermo Scientific Medifuge centrifuge. Light microscope using Omax compound microscope 40x-800 × with objective lens of × 4 and × 10 was used to observe the slides from the histopathological study.

### Methods

#### Characterization of BO using Gas Chromatography-Mass Spectrometry (GC–MS)

The GC–MS analysis was carried out on a PerkinElmer Clarus@ 580 chromatograph that was linked to a mass detector. DMSO-d6 was used to dissolve the samples. One (1.0) µl of a split ratio of 20:1 was injected. The following were the GC runtimes:

lower-alpha initial temperature of 100 °C for 2 min,
1 ramp of 10 °C/min up to 200 °C and hold for 0 min,
2 gradient of 5 °C/min up to 280 °C and hold for 15 min,
3 injection temperature of 250 °C

Helium was used as the carrier gas. MS phase had the following parameters: an ion source temperature of 220 °C, a scan of 50–500 Da in positive ion mode, a solvent delay of 3 min, and a transfer temperature of 280 °C. For data processing and structural prediction, the TurboDEFAULT.PRO software integrated with the NIST Mass Spectral library was used [27].

#### Antibiotics treatment protocol

Antibiotics comprising vancomycin, metronidazole, ampicillin and neomycin (VMAN) with the inclusion of antifungal Amphotericin-B were used in this protocol. Mice were randomized into seven groups (*n* = 7), and given either a single antibiotic (thus either vancomycin, metronidazole, ampicillin or neomycin) plus Amphotericin-B or a cocktail antibiotic (VMAN) plus Amphotericin-B or no antibiotic for a period of 21 days; drugs were administered twice daily [46]. Prior to the 21-day treatment, Amphotericin-B was administered for 3 days bid, and then continued throughout treatment period. Oral gavaging was employed in delivering precise doses of antibiotics. Antibiotic doses were as follows: Ampicillin 1 mg/kg, vancomycin 50 mg/kg, neomycin 100 mg/kg, metronidazole 100 mg/kg, amphotericin-B 1 mg/kg [26, 46]. Control mice received no treatment at all. During the treatment period, the body weight of each animal was checked after every seven days and on the last day of treatment [55].

#### PTZ test post antibiotics treatment

On the 25^th^ day, mice treated with VMAN + Ampho-B were randomly divided into six groups. Groups 1 to 3 received 30, 100 and 300 mg/kg body weight (p.o.) of BO respectively. Groups 4 to 6 (positive control) received diazepam at dose of 0.1, 0.3 and 1 mg/kg body weight (i.p.) respectively. The negative control (mice that received no antibiotic or antifungal treatment) received 25 ml/kg body weight of normal saline p.o. Mice that were treated with single antibiotics + Ampho-B received BO at dose 300 mg/kg. One hour after BO administration, 70 mg/kg of PTZ was administered intraperitoneally to each mouse in all other groups, except for mice in the diazepam group that received PTZ after 30 min. [32]. Mice were observed for 30 min for the presence of seizures.

#### Colon Haematoxylin–Eosin (H&E) staining

Euthanization of mice was achieved using the cervical dislocation technique without prior anaesthesia by a trained technician [31] as this method is less likely to influence the composition of blood and tissues [32]. After euthanizing mice, blood was collected, abdomen dissected and colon removed and washed with 0.9% saline. The colon tissue was cut and fixed in 10% formalin for H&E staining. The stained sections were observed and photographed under a light microscope with magnification of × 10 and × 40 [55].

#### NMR Metabolomics of serum and stool sample

The NMR experiments were performed with a Bruker AscendTM 500 MHz spectrometer (Bruker Biospin GmbH, Germany). The proton frequency of the used spectrometer was set to 500 MHz. The temperature stability was calibrated to be at exactly 298 K. The parameters used are as follows:

- Temperature: 25 °C,
- Acquisition time: 4 s,
- Mixing time: 0.1 s,
- Initial delay: 0.01 s,
- Pre-saturation delay: 0.99 s,
- Transients/scans: 32,
- Steady state scans: 4 and

Spectral width: 12 ppm. Prior to data acquisition, sample were filtered through a 3 KDa Filter Tube. Internal standards employed were 3-(Trimethylsilyl)-1- propanesulfonic acid-d6 sodium salt and Sodium Azide dissolved in Deuterium oxide [22].

##### Metabolite identification

Metabolites were identified by comparing them to a library of reference compounds (in Chenomx NMR Suite Professional) (Chenomx Inc., Edmonton, Canada).

#### Faecal genomic bacterial DNA extraction

Mice faeces were collected and immediately frozen in liquid nitrogen, and then stored at a temperature of -80 °C. The genomic DNA was extracted from approximately 100 mg of faecal samples in 2 ml screw-capped tubes using Quick-DNA™ fungal/Bacterial Miniprep Kit from Zymo Research with slight modifications. Prior to extraction, stool samples were washed and suspended in 500 µl of 1 M phosphate buffer saline (PBS) solution with pH of about 7.4. Two hundred (200) µl of the suspension was aliquoted into a ZR BashingBead™ Lysis Tube (0.1 mm & 0.5 mm) tube and 750 µl of BashingBead™ added to the tube. The tube was then secured in a bead beater and processed at maximum speed for ≥ 5 min. After, tubes were spun at 10,000 g for one minute in a microcentrifuge, 400 µl of the supernatant was transferred onto a Zymo-SpinTM III-F Filter in a collection tube, and the tubes were then spun at 8,000 g for one minute. The filtrate from the aforementioned procedure was added to 1,200 µl of Genomic Lysis Buffer in the collection tube. The mixture was divided into 800 µl, placed in a collecting tube with a Zymo-SpinTM IIC Column, and spun at 10,000 g for one minute.

After discarding the flow via the collection tube, the procedure described above was immediately repeated. A new collection tube was filled with 200 µl of DNA Pre-Wash Buffer, and the Zymo-SpinTM IIC Column was then spun at 10,000 g for one minute in a microcentrifuge. The Zymo-SpinTM IIC Column was filled with 500 µl of g-DNA Wash Buffer and spun at 10,000 g for one minute. Thirty five (35) µl of DNA Elution Buffer and the Zymo-SpinTM IIC Column were added to a clean 1.5 ml microcentrifuge tube before centrifuging at 10,000 g for 30 s. Genomic DNA was eluted at a volume of 100 µl in accordance with the manufacturer’s procedure. DNA was quantified using NanoDrop ND-1000 Spectrophotometer [18].

#### Amplification of the 16S rRNA Gene using RT-PCR

To tag each PCR product, the 16S rRNA gene, which consists of the V3-V5 regions, was amplified. With the aid of a forward and a reverse primer with a special base barcode (10), DNA was recovered from the faeces. A PCR mix of 50 µl was created for each sample. The mix was made up of a 25 ng DNA template, 0.3 µM primer pairs, 10 mM deoxynucleotide triphosphate (dNTP) Mix, 1 U DNA Polymerase and its corresponding buffer. The PCR reaction conditions were configured to include a 95 °C initial denaturation phase for three minutes, afterwards 25 cycles of 98 °C denaturation for 20 s, 45 °C annealing for 15 s, and 72 °C extension for 15 s. The last extension lasted one minute at 72 degrees. The primer pairs included the forward primer (5′-GCCTTGCCAGCCCGCTCACTCCTACGGGAGGCAGCAG-3′), which was a combination of primer 454B and primer 338F, and the reverse primer (5′-GCCTCCCTCGCGCCATCAGNNNNNNNNNNCCGTCAATTCMTTTGAGTTT-3′), also a combination of primer 454A, a special 10 base barcode and a 907R primer. PCR amplicons were purified by means of the QiaQuick PCR Purification Set after replicate PCRs were pooled (Qiagen, USA) [8].

#### 16S rRNA gene sequencing and bioinformatics analysis

Following the recommendations of 454 Roche, the amplicons from the PCR were sequenced by means of a 454 FLX pyro sequencer platform. All effective reads were grouped into operational taxonomic units (OTUs) based on 97 percent sequence similarity which was in accordance with the 454 standard of operation. Reads high in quality were chosen for bioinformatics analysis. High quality raw readings were not selected for sequences that did not have the V3-V5 primers, had a short variable area of less than 90 bp, or had an unknown nucleotide in the V3-V5 variable region. For sequence trimming, the de-noising criteria comprised a quality score of 35 (averagely) within the 50 base pair window and the use of a 50 base pair sliding window to minimise error due to sequencing. In SILVA-compatible database alignment, all high quality sequences were aligned using the closest alignment space termination multi-aligner. Inconsistent reads were eliminated. Using a brand-new Ribosomal Database Project, a Bayesian classifier was used to categorize each read. Additionally, reads that could not be categorised at the kingdom level were excluded. The Shannon index and Alpha diversity analysis were calculated using Quantitative Insights into Microbial Ecology (QIIME). Principal component analysis (PCA) was performed using Fast UniFrac. The representative of each OTU was obtained by means of QIIME and was included in the phylogenetic diagram. According to well-known published statistical methods, the statistical significance of group separation in PCA score plots was determined using a multivariate analysis of variance test for statistical significance. ANOVA was used to analyse normally distributed data using Tukey's post hoc test. Redundancy analysis models were built using the relative abundance of each OTU. According to the manufacturer's recommendations, Canoco for Windows 4.5 was used to test OTUs that varied between groups. The Monte Carlo Permutation Procedure was used to assess statistical significance using 499 random permutations [2, 8].

#### Data analysis

One-way analysis of variance (ANOVA) was conducted to determine whether there were any significant differences among the group means. To identify specific pairwise differences among the group means that exhibited significant differences in the ANOVA, Tukey's multiple comparison test was conducted. To investigate the impact of various treatments and depletion of microbiota on seizure indicators, a Two-way Analysis of Variance (ANOVA) followed by Tukey's multiple comparisons test was conducted. Data were presented as mean ± standard error mean (S.E.M). GraphPad Prism® Version 8.0.1 (GraphPad Software, CA, USA) was used for statistical analyses. In all cases, *P* < 0.05 was considered significant.

## Results

### Characterization of BO using GC–MS

**Fig. 1:**
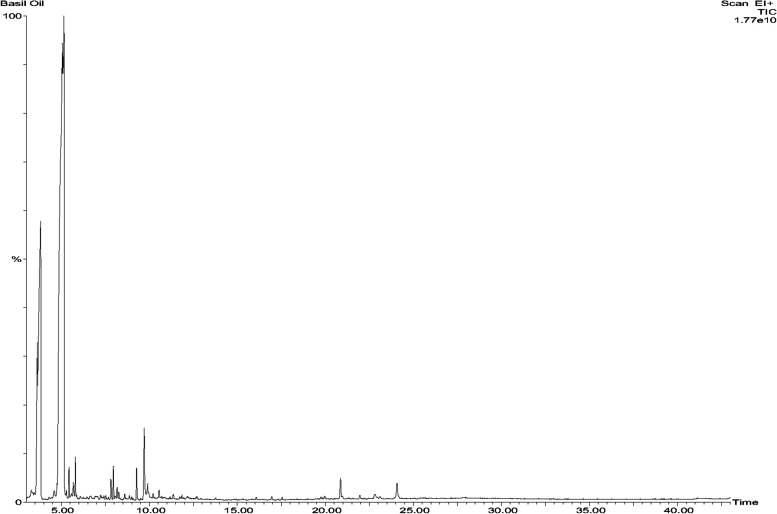
GC–MS chromatogram of BO showing peaks of the constituents of BO at various retention times

In the GC–MS analysis of BO, linalool had a retention time of 3.835 with an area percentage of 21.83% (Table 1) while estragole had a retention time of 5.121 with an area percentage of 58.12% (Table 1). These two compounds appear to be major components in BO. GC–MS chromatogram predicted several minor components of BO (Fig. 1).

**Table 1:** GC–MS analysis of BO detailing the retention times, height, area and normalization of the constituents

No	Names	Retention time	Height (cm)	Area %	Norm %
1	α-methyl-α-[4-metyl-3-pentenyl]oxiranemethanol	3.269	275,817,924	0.43	0.772
2	Linalool	3.835	10,123,077,709	21.83	37.63
3	12-oxabicyclo[9.1.0]dodeca-3,7-diene	4.591	302,821,415	0.4	0.699
4	Estragole	5.121	17,567,600,910	58.12	99.7
5	β- Citral	5.439	1,123,603,601	0.394	0.675
6	Benzaldehyde, 4-methoxy	5.685	521,998,167	0.282	0.485
7	2,6,octadienal,3,7-dimethyl-(E)	5.801	1,441,309,201	0.525	0.912
8	Caryophyllene	7.798	721,122,706	0.392	0.66
9	2-Norpinene	7.951	1,178,605,811	0.543	0.959
10	1,4,7-cycloundecatriene,1,5,9,9-tetramethyl-ZZZ-	9.266	1,181,715,202	0.644	1.1
11	Trans-4-methoxycinnamaldehyde	9.72	2,602,580,048	1.811	3.3
12	Caryophyllene oxide	9.894	518,706,555	0.614	1.046
13	Spiro[4,5]decan-7-one,1,8-dimethyl-8,9-epoxy-4-isopropyl	12.153	107,278,570	0.234	0.443
14	1-butanone,2-chloro-3-methyl-1-[4-(1-methyl ethyl)phenyl]	20.868	772,139,790	0.621	1.139
15	4-(cis-2,3,4,trans-6-tetramethyl-3-cyclohexenyl) butan-2-one,2,4-dinitrophenyl hydrazone	22.798	150,916,175	0.23	0.443
16	1-butanone,2-chloro-3-methyl-1-[4-(1-methyethyl) phenyl]	24.079	575,736,922	0.762	1.311
17	1-monolinoleoylglycerol trimethylsilyl ether	27.591	44,897,334	0.308	0.53
18	1-monolinoleoylglycerol trimethylsilyl ether	27.783	55,845,643	0.3625	0.61
19	Trilinolein	41.128	53,311,625	0.3185	0.52

### Pentylenetetrazole (PTZ) Test

**Fig. 2:**
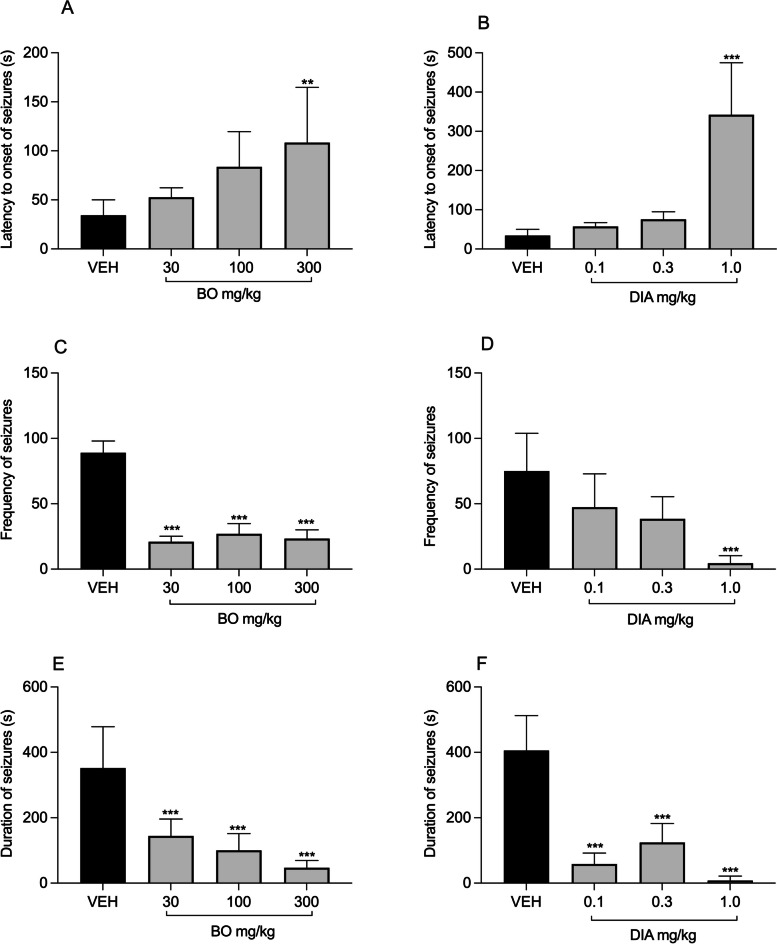
Effects of BO (30, 100 and 300 mg/kg, p. o.); and diazepam (0.1, 0.3 and 1 mg/kg, i.p.); on the latency (**A**, **B**), frequency (**C**, **D**) and duration (**E**, **F**) of seizures induced with PTZ. Data are presented as group mean ± SEM (*n* = 7). ** *P* < 0.01, ***P < 0.001 compared with vehicle treated group (One-way analysis of variance followed by Tukey's multiple comparisons test)

BO at dose 300 mg/kg [*P* = 0.00071; F (8, 20) = 5.386; Fig. 2A] and the standard drug DIA at 1 mg/kg significantly [*P* < 0.0001; F (3, 17) = 31.52; Fig. 2B] reduced the latency to onset of seizure. Also, BO significantly reduced the frequency [*P* < 0.0001; F (3, 14) = 76.56; Fig. 2C] and the duration [*P* < 0.0001; F (3, 22) = 20.41; Fig. 2E] of seizures similar to the standard DIA (Fig. 2D and F).

### PTZ test post-antibiotic treatment

#### Cocktail antibiotic treatment

**Fig. 3:**
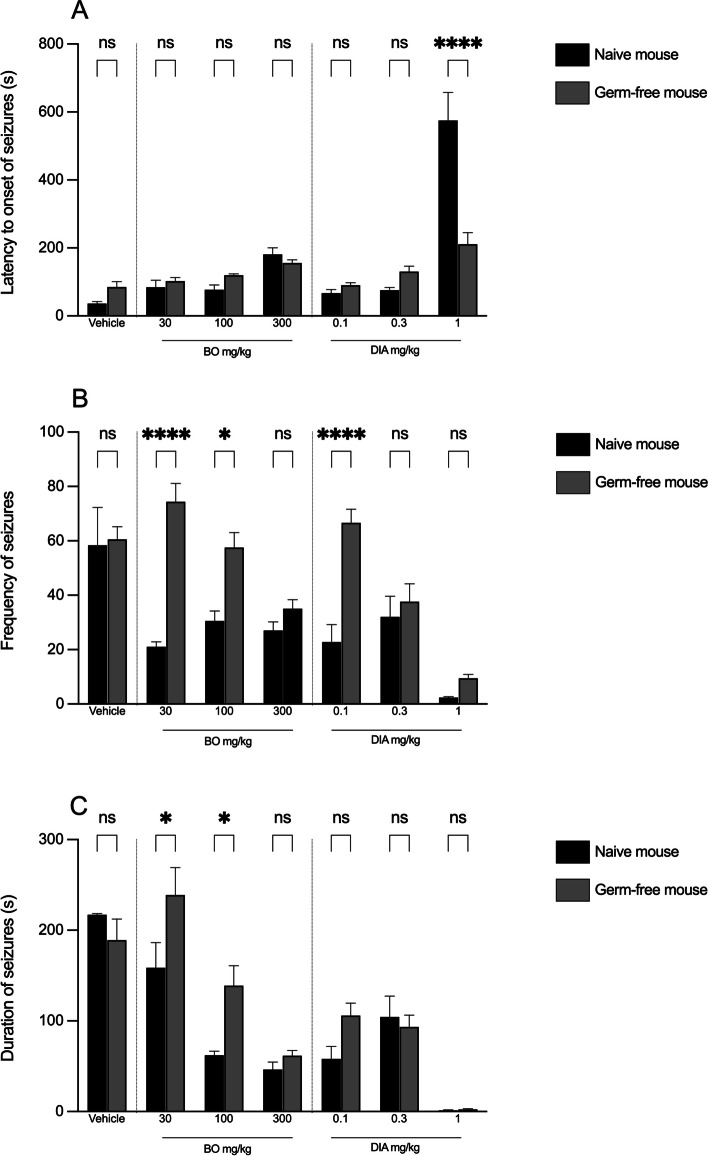
Antiseizure effects of BO (30, 100, 300 mg/kg, p. o.) and DIA (0.1, 0.3, 1 mg/kg, i. p.) in naïve mouse (no antibiotic treatment) and germ-free mouse (treated with VMAN). Assessment was based on seizure latency (**A**), frequency (**B**), and duration (**C**) induced by PTZ. The antiseizure effects were reversed following antibiotic pre-treatment. The presented data display the mean of the respective groups ± SEM (*n* = 7). * indicates a significance level of *P* < 0.05, while **** indicates *P* < 0.0001 (assessed through Two-way ANOVA followed by Tukey's multiple comparisons test)

Treatment of mice with cocktail antibiotics significantly [*P* = 0.0289; F (1, 56) = 5.032; Fig. 3A] reversed action of DIA (1 mg/kg) against latency to onset of convulsions. The effects on latency for all other treatments were not significantly affected. Effects of BO at 30 mg/kg [*P* < 0.0001; F (1, 58) = 40.16; Fig. 3B] and 100 mg/kg [*P* < 0.0001; F (1, 58) = 40.16] against frequency of seizures were significantly reversed after cocktail antibiotic pre-treatment. Similarly, effect of DIA against frequency of seizures was reversed at dose of 0.1 mg/kg. Effect of BO against duration of seizures was significantly reversed at doses 30 mg/kg [*P* = 0.0129; F (1, 64) = 6.548; Fig. 3C] and 100 mg/kg [*P* = 0.0129; F (1, 64) = 6.548; Fig. 3C]. All other treatments were not significantly affected by antibiotic pre-treatment.

#### Single antibiotic treatment

**Fig. 4:**
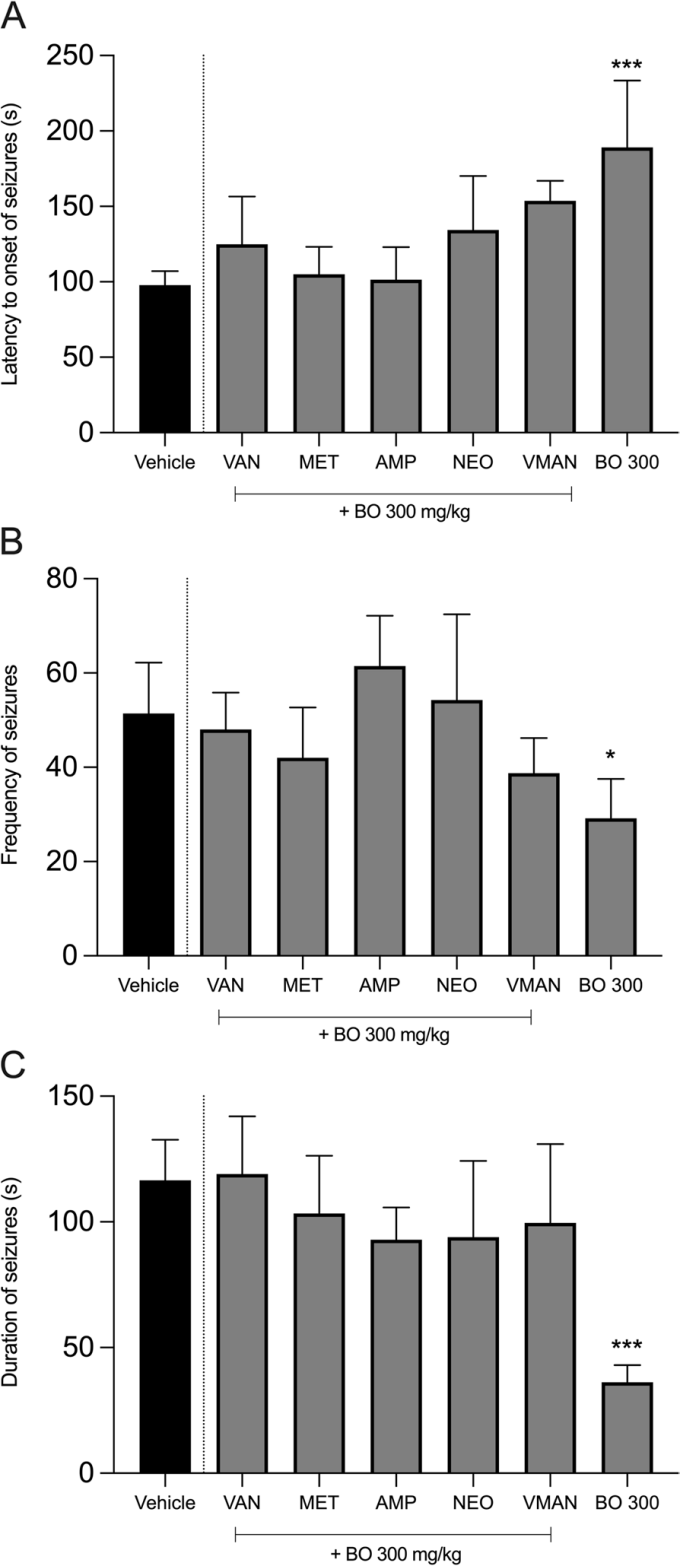
Antiseizure effects of BO at 300 mg/kg in mice pre-treated with single antibiotics (van, met, amp, and neo) compared to BO at 300 mg/kg in naive mice (no antibiotic treatment). Assessment was based on seizure latency (**A**), frequency (**B**), and duration (**C**) induced by PTZ. The antiseizure effects were reversed following antibiotic pre-treatment. The data are shown as group mean ± SEM (*n* = 7). * denotes *P* < 0.05, *** denotes *P* < 0.001 (one-way analysis of variance followed by Tukey's multiple comparisons test)

In mice that were pre-treated with single antibiotics, effect of BO at dose 300 mg/kg was lost against latency to seizures (Fig. 4A), frequency (Fig. 4B) and the duration (Fig. 4C) of seizures compared to mice that received BO at 300 mg/kg only without antibiotic pre-treatment.

### Metabolomics analyses of serum samples post antibiotic treatment

**Fig. 5:**
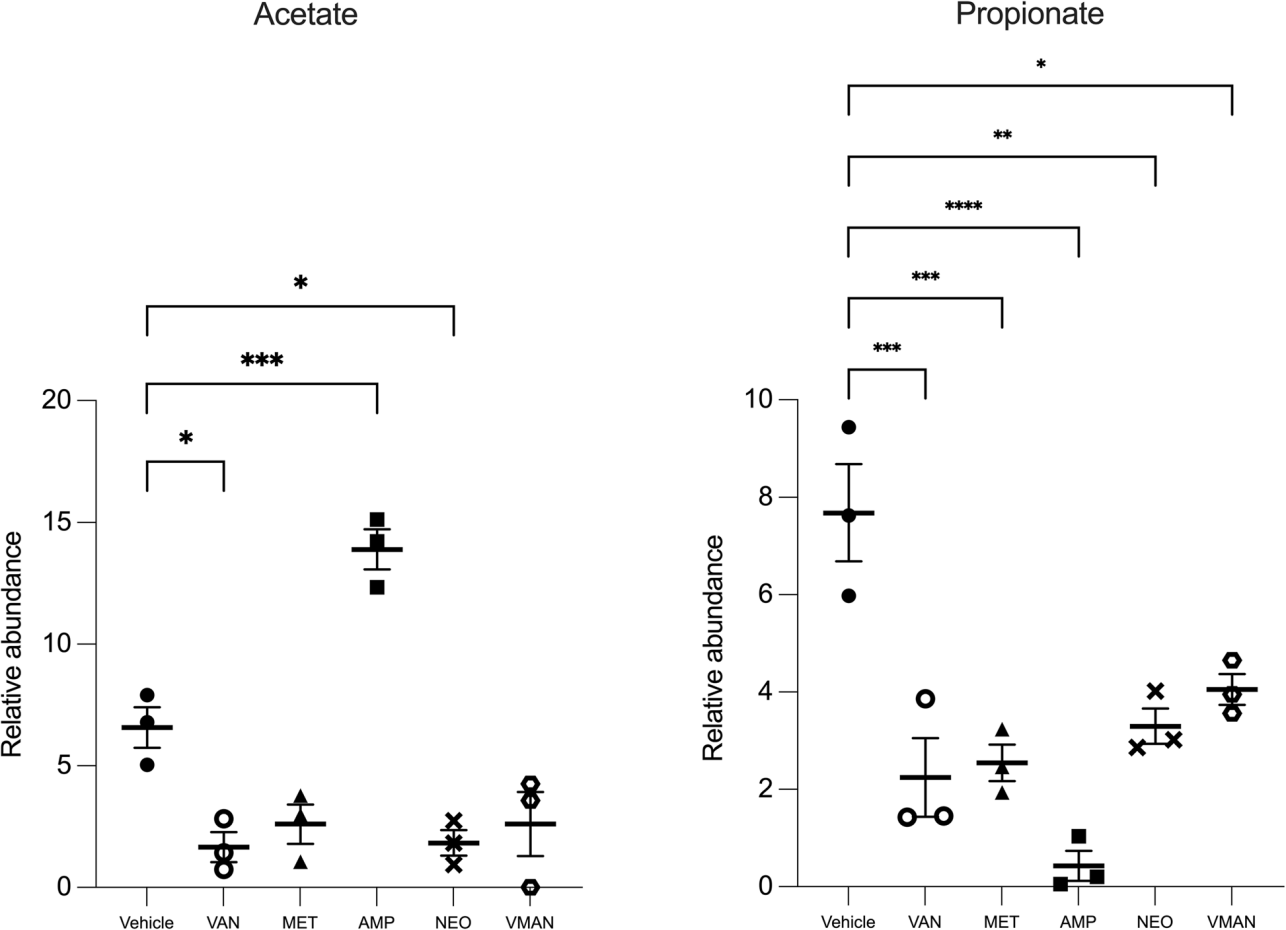
Relative abundance of acetate and propionate in serum of mice pre-treated with VMAN, van, met, neo, amp and the vehicle group. Metabolites were analysed using 1H NMR-based metabolomics. Acetate and propionate were the most abundant metabolites. The values are presented as mean ± SEM (*n* = 3). * denotes *p* < 0.05, ** *p* < 0.01, *** *p* < 0.001, **** *p* < 0.0001

In the untargeted 1H NMR of the serum sample, the peaks of 22 metabolites giving strong and distinct signals in NMR spectra were chosen for the analysis. The SCFAs identified were acetate and propionate. Other metabolites such as alanine, creatine, glutamate, glutamine, glycine, gluconate, glycerol, isoleucine, histidine, phenylalanine, succinate, taurine, valine, lactate and tyrosine were detected in varying concentrations. From Fig. 5, mice pre-treated with ampicillin recorded the highest relative abundance of acetate (*P*<0.001). However, there was no significant difference in the relative abundance of acetate in mice pre-treated with VMAN as compared to the vehicle. The relative abundance of propionate in all treatment groups were significantly lower compared to the vehicle group. Also, another observation from the serum metabolomics was the high concentrations of glutamine (14.34 mM ± 0.77) and glutamate (12.68 mM ± 0.64) and corresponding low concentration of glycine (7.83 mM ± 0.95) in the VMAN treated group compared to the vehicle group [glutamine (5.58 mM ± 1.56), glutamate (6.83 mM ± 1.34) and glycine (14.43 mM ± 0.11)]. The vancomycin treated group exhibited a similar metabolic signature as compared to the VMAN group with the presence of high concentrations of glutamine (9.37 mM ± 1.27), glutamate (13.93 mM ± 1.46), and low concentrations of glycine (0.82 mM ± 0.02) in the serum. The scatter plot in Fig. 5 shows the relative abundance of acetate and propionate in molar concentrations in the serum of the various pre-treatment groups. The ascription of signals used for the metabolite quantification in the NMR spectra is shown in Table 2.

**Table 2:** 1H NMR metabolomics of serum sample

Metabolite (Serum)	Vehicle (mM)		Van (mM)		Met (mM)		Amp (mM)		Neo (mM)		VMAN (mM)	
	Conc	S.E.M	Conc	S.E.M	Conc	S.E.M	Conc	S.E.M	Conc	S.E.M	Conc	S.E.M
Alanine	ND	ND	13.19	1.06	ND	ND	1.85	1.56	1.87	0.68	5.96	0.71
Creatine	ND	ND	0.14	0.09	0.35	0.05	0.02	1.57	0.48	0.29	5.72	0.34
Ethanol	5.07	0.18	4.69	1.54	ND	ND	1.29	1.1	0.23	1.41	12.51	0.54
Gluconate	5.03	0.01	0.05	0.07	2.91	0.04	1.34	0.64	ND	ND	4.38	0.76
Glucose	9.12	0.03	6.07	0.88	ND	ND	ND	ND	ND	ND	6.89	0.63
Glutamate	6.83	1.34	13.93	1.46	ND	ND	ND	ND	3.77	0.06	12.68	0.64
Glutamine	5.58	1.55	9.37	1.27	ND	ND	ND	ND	3.76	0.65	14.34	0.77
Glycerol	10.55	0.81	3.60	0.61	1.02	0.62	ND	ND	0.61	0.62	15.25	0.08
Glycine	4.43	0.11	0.82	0.02	ND	ND	ND	ND	1.19	0.98	7.83	0.95
Histidine	7.85	0.78	62.74	0.07	ND	ND	0.27	0.00	ND	ND	1.74	1.58
Lactate	30.24	0.28	59.13	0.64	ND	ND	1.77	1.23	ND	ND	3.89	0.2
Leucine	ND	ND	48.01	1.11	ND	ND	3.32	0.58	ND	ND	13.64	0.34
Phenylalanine	17.67	1.27	15.46	1.33	0.46	0.00	ND	ND	ND	ND	ND	ND
Pyruvate	1.965	0.99	ND	ND	ND	ND	ND	ND	1.98	1.54	4.21	1.38
Serine	7.452	0.00	35.27	1.15	ND	ND	ND	ND	ND	ND	12.21	0.29
Succinate	0.956	1.16	ND	ND	ND	ND	ND	ND	ND	ND	ND	ND
Taurine	1.73	0.021	2.31	0.05	0.93	0.17	ND	ND	ND	ND	ND	ND
Tyrosine	17.32	1.37	15.75	1.43	0.37	0.02	0.67	0.40	ND	ND	ND	ND
Valine	7.03	1.05	8.11	0.4	ND	ND	1.30	0.61	ND	ND	ND	ND

*ND* Not detected

#### Stool metabolomics

**Fig. 6:**
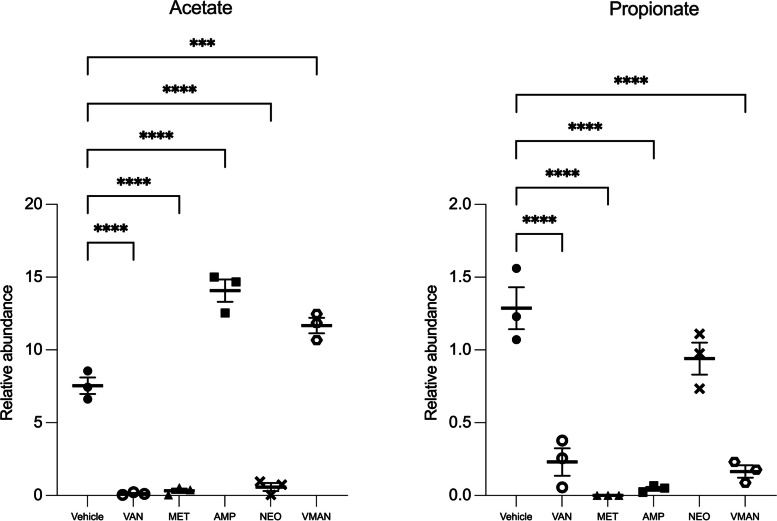
Relative abundance of acetate and propionate in stool samples of mice pre-treated with VMAN, van, met, amp, neo and the vehicle group. Metabolites were analysed using 1H NMR-based metabolomics. Acetate and propionate were the most abundant metabolites. The values are stated as mean ± SEM (*n* = 3). *** denotes *P* < 0.001, **** denotes *P* < 0.0001

The 1H NMR detected about 14 metabolites with strong and distinct signals (Table 3). Similarly, acetate and propionate were detected in all samples. Comparable to the serum metabolomics analysis, the relative abundance of acetate in the ampicillin pre-treated group was significantly (*P*<0.0001) higher than in the vehicle treated group. VMAN pre-treated group also recorded a significantly (*P*<0.001) higher relative abundance than the vehicle group. Acetate abundance in all other groups was significantly (*P*<0.0001) lower compared to the vehicle group. Furthermore, the relative abundance of propionate in any other treated group was significantly (*P*<0.0001) lower compared to the vehicle group. Here, glutamine, glutamate and glycine were only detected in the vancomycin pre-treated group. Figure 6 is a scatter plot showing the concentrations of acetate and propionate in stool metabolomics of mice.

**Table 3:** 1H NMR metabolomics of stool sample

Metabolite (Stool)	Vehicle (mM)		Van (mM)		Met (mM)		Amp (mM)		Neo (mM)		VMAN (mM)	
	Conc	S.E.M	Conc	S.E.M	Conc	S.E.M	Conc	S.E.M	Conc	S.E.M	Conc	S.E.M
Alanine	ND	ND	ND	ND	ND	ND	2.72	0.27	1.53	0.06	6.03	0.96
Creatine	2.37	0.13	1.89	0.49	0.11	1.19	1.61	0.47	0.47	0.39	1.65	0.02
Ethanol	ND	ND	ND	ND	ND	ND	ND	ND	6.33	1.36	13.56	1.51
Gluconate	9.68	1.44	3.26	0.88	1.43	0.88	ND	ND	0.79	1.03	7.46	0.24
Glucose	ND	ND	8.45	1.38	0.71	0.20	ND	ND	0.49	1.10	5.34	1.37
Glutamate	ND	ND	7.53	0.72	ND	ND	ND	ND	ND	ND	ND	ND
Glutamine	ND	ND	6.61	1.00	ND	ND	ND	ND	ND	ND	ND	ND
Glycerol	ND	ND	ND	ND	1.13	1.39	ND	ND	ND	ND	ND	ND
Glycine	ND	ND	ND	ND	0.05	0.65	ND	ND	ND	ND	ND	ND
Histidine	ND	ND	7.07	0.36	ND	ND	ND	ND	ND	ND	ND	ND
Isoleucine	ND	ND	ND	ND	2.68	1.53	ND	ND	ND	ND	10.45	1.10
Lactate	0.15	0.08	ND	ND	ND	ND	ND	ND	ND	ND	0.68	0.77
Leucine	ND	ND	4.29	0.41	ND	ND	ND	ND	5.68	0.89	10.68	0.31
Phenylalanine	ND	ND	0.04	0.00	0.32	0.55	ND	ND	0.77	0.71	2.54	0.12
Pyruvate	0.21	0.01	ND	ND	0.09	0.90	0.88	1.23	0.32	0.39	0.67	1.16
Serine	ND	ND	ND	ND	1.04	0.35	ND	ND	ND	ND	ND	ND
Succinate	ND	ND	ND	ND	0.38	1.57	0.43	1.39	0.54	1.53	0.03	1.06
Tyrosine	ND	ND	ND	ND	3.43	1.32	ND	ND	ND	ND	ND	ND
Valine	ND	ND	ND	ND	0.096	1.05	1.28	0.02	0.52	0.49	ND	1.5741

#### Metabolite clustering

**Fig. 7:**
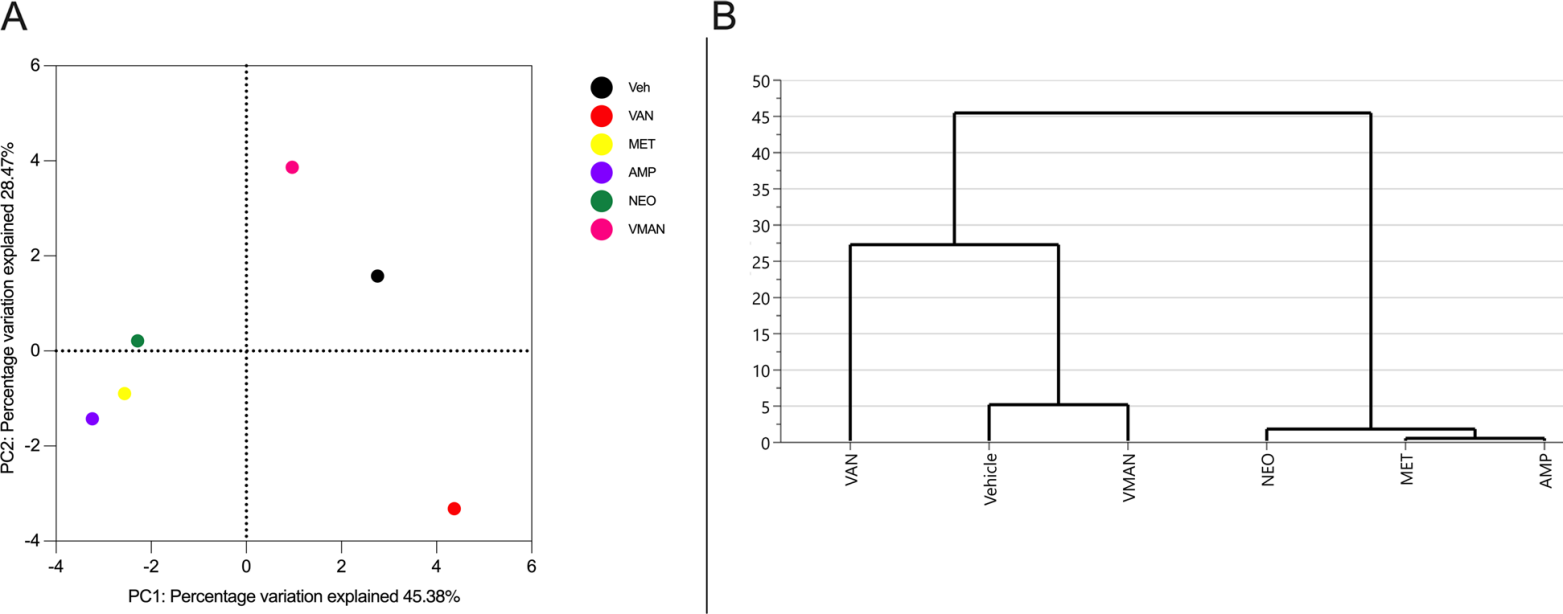
**A** Principal components analysis (PCA) score plots of metabolites in serum of naïve mice (veh), vancomycin pre-treated (van), metronidazole pre-treated (met), ampicillin pre-treated (amp), neomycin pre-treated (neo) and the cocktail (VMAN) pre-treated mice. The points on the plot correspond to the metabolic profile of the respective groups. **B** Hierarchical clustering of multivariate analysis of variance from PCA matrix scores. The clustering identifies whether the metabolite compositions of the various pre-treated mice groups exhibit distinctive grouping or similar patterns

Multivariate analysis of variance of PCA matrix scores (Fig. 7A) indicated a statistically significant separation between the metabolites detected in serum of vehicle treated and VMAN, van, neo, amp and met pre-treated groups (Fig. 7B). Metabolites in serum of mice pre-treated with met and amp were similar and not significantly different from neo pre-treated mice.

### Faecal genomic bacterial DNA extraction

Pure genomic bacteria DNA was extracted and quantified using the nanodrop spectrophotometer. The concentration of DNA in each sample (ng/ul) is shown in Table 4. Assessment of the purity of the DNA based on the ratio of absorbance at 260 nm (A260) to absorbance at 280 nm (A280) shows absorbance ratios above 1.8 indicating the absence of other proteins or impurities.

**Table 4:** Nanodrop quantification and purity determination of extracted DNA from stools of mice pre-treated with VMAN, the single antibiotics and the vehicle group

Sample	DNA conc. (ng/ul)	260/280 absorbance ratio
Vehicle	389.15 ± 4.38	1.89 ± 0.00
VMAN	485.00 ± 1.48	1.88 ± 0.00
Van	429.86 ± 1.40	1.87 ± 0.00
Met	379.95 ± 4.33	1.87 ± 0.00
Amp	420.48 ± 4.63	1.87 ± 0.00
Neo	345.53 ± 2.71	1.87 ± 0.00

The values are shown in mean ± SEM (*n* = 3)

### Bacterial taxa classification

**Fig. 8:**
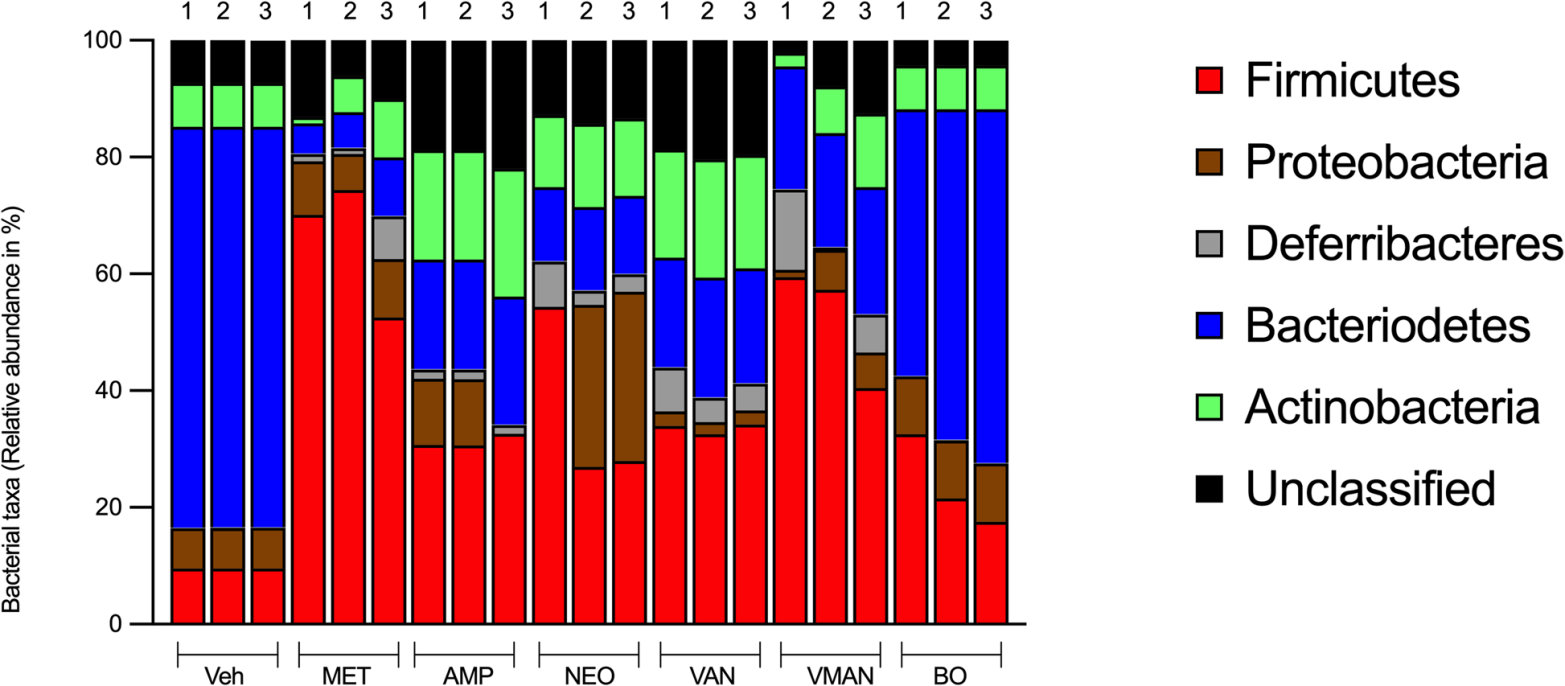
Relative abundance of phyla of microorganisms identified from the sequence reads. DNA was obtained from faecal matter of mice in the naïve group (veh), and mice pre-treated with metronidazole (met), Ampicillin (amp), neomycin (neo), vancomycin (van), cocktail of antibiotics (VMAN) and basil oil (BO)

After PCR and High-throughput sequencing, a total of six (6) bacterial phyla were detected at the phylum level with 1 phylum remaining unclassified (Fig. 8). The detected five (5) phyla are *Firmicutes, Proteobacteria, Deferribacteres, Bacteroidetes* and *Actinobacteria*. Samples were dominated by *Bacteroidetes* and *Firmicutes*. The percentage abundance of *Bacteroidetes* in the vehicle group was 70%, *Firmicutes* 10%, *Proteobacteria* 6*%, Actinobacteria* 7%, unclassified phylum 7%. The VMAN pre-treatment group recorded a relative abundance of *Bacteroidetes* of 20%, *Firmicutes* 52.7%, *Proteobacteria* 3.7%, *Actinobacteria* 6%, *Deferribacteres* 6.7% and the unclassified phylum with about 6.7%. The metronidazole group showed an abundance of *Firmicutes* at 65.3%, *Bacteroidetes* 5%, *Actinobacteria* 3%, *Deferribacteres* 2.7% and unclassified 9.3%. The relative abundance of phyla in the ampicillin and vancomycin group were similar. Notably, the taxonomic profile of the BO-only treated group was similar to the vehicle group with relative abundance of *Bacteroidetes* of 51%, *Firmicutes* of 24%, *Proteobacteria* of 12%, and *Actinobacteria* of 8% and the unclassified phylum of 5%. Bacteria identified were *Deferribacteraceae sp., Lachnospiraceae sp., Enterobacteriaceae sp., Enterococcaceae sp., Erysipelotrichaceae sp., Ruminococcaceae sp., Eubacteriaceae sp., Streptococcaceae sp., Lachnospiraceae sp., Paenibacillaceae sp., Ruminococcaceae sp., Staphylococcaceae sp., Erysipelotrichaceae sp., Coriobacteriaceae sp., Corynebacteriaceae sp., Lactobacillaceae sp., Eubacteriaceae sp.,*

### Microbiome clustering

**Fig. 9:**
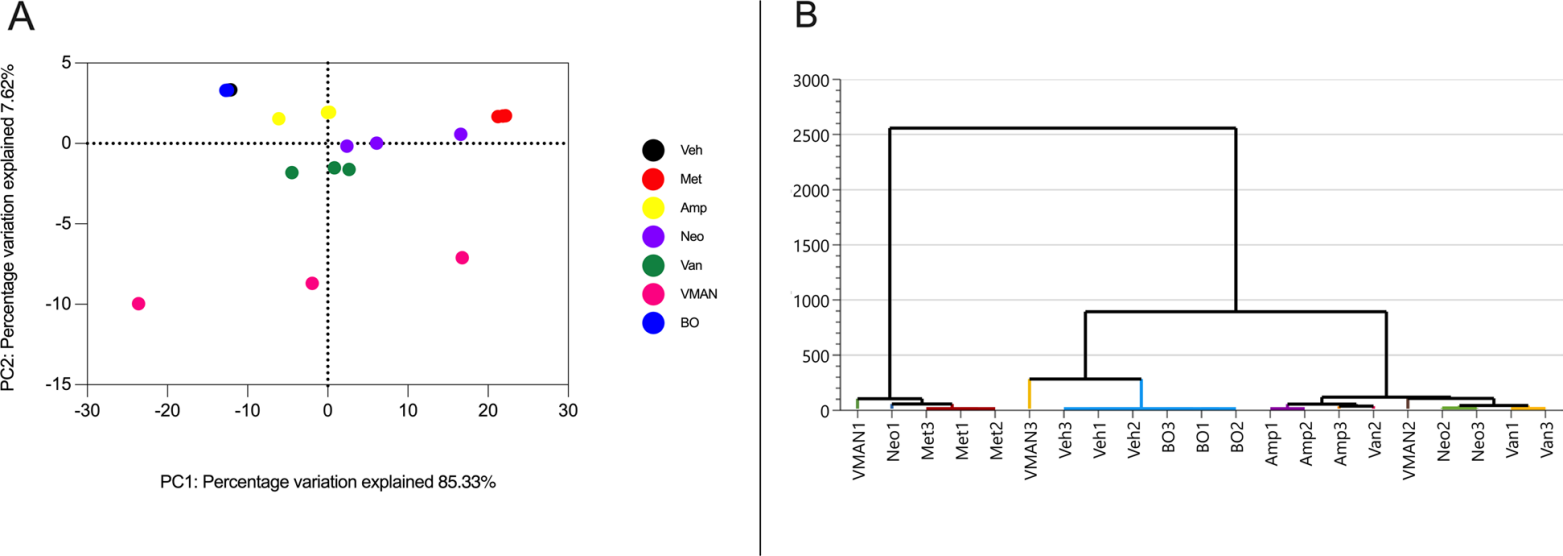
**A** Principal components analysis (PCA) score plots of microbiome in faecal matter of naïve mice (veh), and mice pre-treated with metronidazole (met), Ampicillin (amp), neomycin (neo), vancomycin (van), basil oil (BO) and the cocktail of antibiotics (VMAN). The points on the plot correspond to the microbiome profile of the mice in the respective groups. **B** Hierarchical clustering of multivariate analysis of variance from PCA matrix scores. The clustering identifies whether the microbiome compositions of the various pre-treated mice groups exhibit distinctive grouping or similar patterns

Multivariate analysis of variance of PCA matrix scores (Fig. 9A) indicated a statistically significant separation between the microbiota of vehicle treated and VMAN cocktail, van, met, amp and neo, pre-treated groups (Fig. 9B). Significant separations were also noted among VMAN, amp and van pre-treated groups (Fig. 9B). Conversely, the gut microbiota composition of BO-only treated mice did not significantly differ from vehicle treated mice.

### Colon histology

**Fig. 10:**
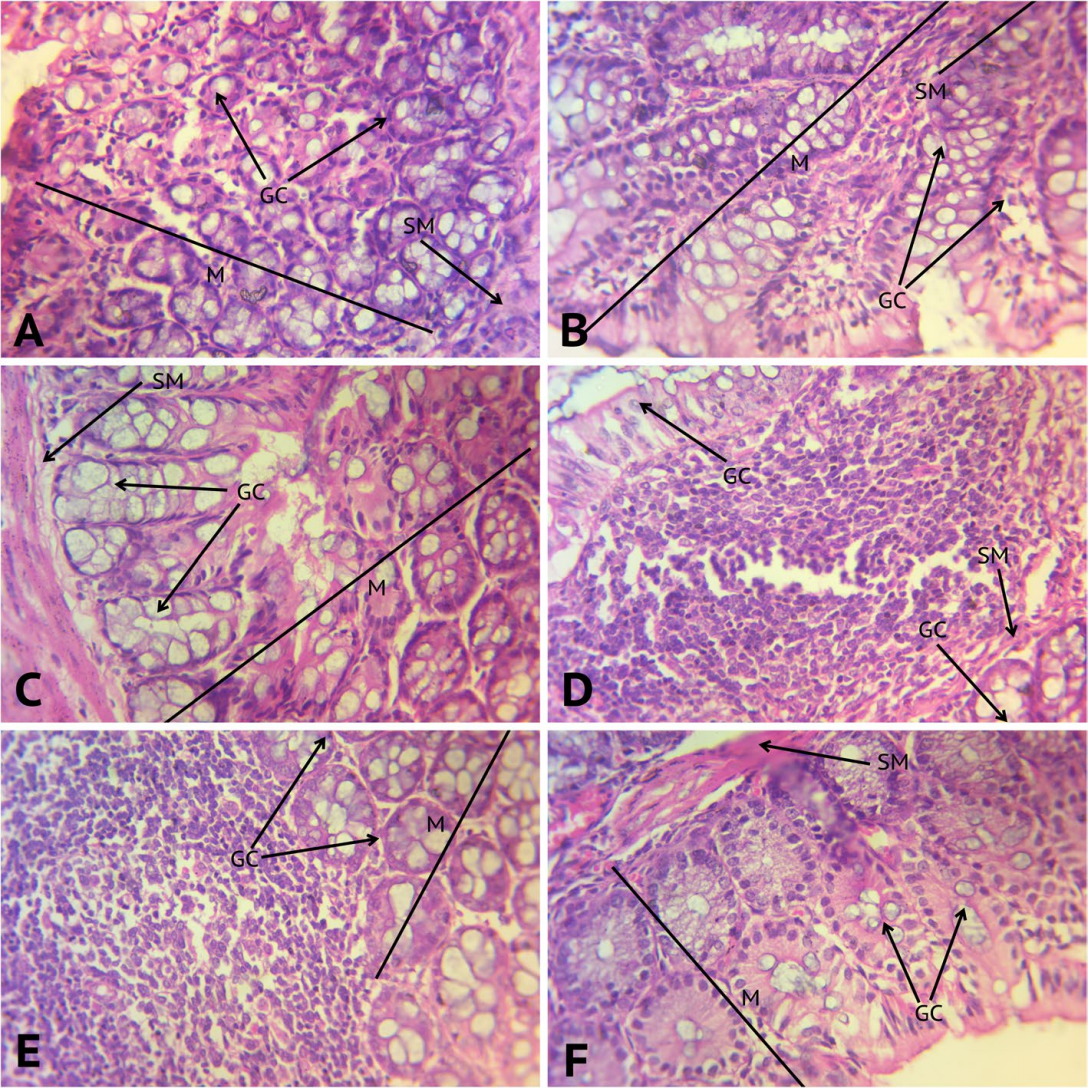
Photomicrograph of H&E stained adult mouse colon. Image **A** showing colon of mice from vehicle group, **B** VMAN group, **C** vancomycin pre-treated group, **D** metronidazole pre-treated group, **E** ampicillin pre-treated group, and **F** neomycin pre-treated group (× 40 magnification). Key: GC, Goblet cells; M, Mucosa; SM, Submucosa

Histopathology of the colon was performed after treatment with VMAN cocktail and also individual antibiotics. Compared to the control, colon of mice treated with VMAN cocktail and the single antibiotics did not show any structural differences (Fig. 10). The goblet cells were normal. Mucosa and submucosa were normal with no inflammation.

## Discussion

The anticonvulsant action of BO was ascertained using the PTZ-induced seizure model. This model has been used extensively to induce seizures in murine models. Depending on dosage, PTZ can produce myoclonic jerky movements, clonic convulsions, and/or forelimbs or hind limbs tonic extensor movements. Also, the PTZ test is known to identify compounds that enhance GABAergic neurotransmission or that act by affecting T-type calcium (Ca^2+^) channels [13, 36, 54]. BO significantly delayed seizure latency, and reduced frequency and duration similarly to standard drug diazepam.

Numerous essential oils have been acknowledged for their anticonvulsive properties. Further studies report the use of the essential oil of *Ocimum basilicum* as an anticonvulsant [30]. Also, previous works have testified to the folkloric use of the leaves and/or smoke of *Ocimum basilicum* in controlling epilepsy [31]. The anticonvulsant activity of most essential oils has been linked to their composition. The essential oil from *Ocimum basilicum*, basil oil, obtained from the aerial part of *Ocimum basilicum* is known to contain linalool, eugenol and cineole as major constituents. These constituents have been reported to be effective in the pentylenetetrazole, picrotoxin, and maximal electroshock seizure models [31, 48, 52].

Linalool, one of the major constituents confirmed by the GC–MS analysis of BO, has been documented to act through inhibition of glutamatergic neurotransmission and through suppression of voltage-gated currents [40]. Knowledge of linalool's properties enhances our understanding of the mechanisms through which this essential oil potentially exerts its anticonvulsant actions. This knowledge not only provides insights into the potential therapeutic applications of basil oil in managing seizures but also contributes to the broader exploration of plant-derived compounds for neurological health.

While investigating the effects of microbiota composition on seizure outcomes, pre-treatment of mice with antibiotics resulted in the depletion of gram-positive, gram-negative, aerobic and anaerobic bacteria in the gut. The combination of VMAN has been known to deplete faecal bacterial DNA load by at least 400 folds without causing mortality [46, 57]. Inclusion of an antifungal Amphotericin-B, was done to prevent fungal overgrowth during treatment, as highlighted from previous studies [28, 46]. The combination of antibiotics and antifungal agents yields a synergistic effect in altering the microbiota. This approach minimizes the chances of microbial resistance, which can develop when a single agent is used over a prolonged period [28, 46]. In this study, depletion and altering of the gut microbiota did not cause any treatment related morbidities. However, reduction in spleen weight in these mice at necropsy has been shown as a characteristic which is phenotypic of germ-free mice [46].

Pre-treatment of mice with cocktail antibiotics significantly reversed the anticonvulsant action of BO and DIA in the PTZ-induced seizure model. The parameters measured, namely latency, frequency and duration of seizures contribute to the overall seizure severity and administration of cocktail antibiotics altered these parameters in varying degrees. Thus, increasing seizure severity. Also, mice that were pre-treated with individual antibiotics exhibited a reversal of the anticonvulsant action of BO. These observations support the claims from previous studies that reiterate the involvement of the microbiota in epilepsy. Bridging the human context with basic experimental findings, the growing body of evidence resonates with the discoveries made in animal models. As emerging research uncovers a potential link between the gut microbiota and epilepsy, notable instances have been documented. A notable case-report described a 22-year-old with Crohn's disease and seizures who achieved seizure remission after a faecal microbiota transplant (FMT), prompting a clinical trial to explore FMT's impact on epilepsy [23]. Additionally, separate studies found that probiotic treatment significantly reduced seizure frequency by over 50% in drug-resistant epilepsy patients [19].

Our observations therefore align with the existing body of knowledge and lend credence to claims from prior research, affirming that a rich and diverse microbiota could potentially alleviate the severity of seizure activity, as corroborated by other studies conducted [3, 15, 14].

From the phylogenetic data, the composition of bacteria in the BO-only treated group was similar to that of the vehicle group with higher a *Bacteroidetes* and a lower *Firmicutes* population. These two phyla constitute the most abundant phyla in the gut existing in a ratio where *Bacteroidetes* are naturally more abundant than *Firmicutes*. The distortion of this balance is believed to cause various diseases [29]. In line with this, mice pre-treated with VMAN presented with increased *Firmicutes* and reduced *Bacteroidetes* (F/B) in a ratio of about 2.5: 1 respectively. From previous studies, an increased F/B ratio has been related to increased frequency of seizure activity while an increase in B/F ratio has been linked to a decrease in seizure activity [9, 24, 60]. Increased seizure activity may account for the reversal of BO anticonvulsant activity observed. In further support of these findings, studies have shown that the gut microbiomes of drug-resistant and drug-sensitive epilepsy patients exhibit disparities. Drug-resistant patients have displayed heightened alpha diversity and increased rare bacteria associated with the *Firmicutes* phylum [43]. Unfortunately, while the taxonomic analysis in many studies have compared patient groups, the extent of deviation from healthy controls remains unexplored, leaving an intriguing question unanswered.

SCFAs are metabolic by-products produced by gut bacteria during dietary fibre fermentation and are believed to play a role in seizure control through various mechanisms. SCFAs have been mentioned to modulate GABAergic neurotransmission and neurotrophic factors thereby exerting anti-neuroinflammatory effects, influencing blood–brain barrier integrity and hence, affecting brain function and development [53]. While the exact connections between SCFAs and seizure control are still being explored, their ability to modulate these pathways suggests a potential link between gut microbiota, SCFAs, and brain function in the context of epilepsy. Acetate, propionate and butyrate are the most clinically important SCFAs. The significant increase in the concentration of acetate in both the stool sample of mice pre-treated with VMAN and ampicillin may not be associated with their increased F/B ratio but may be due to the fact that acetate is the most abundant of the three SCFAs [56]. The increase in acetate concentration in both stool and serum of mice pre-treated with ampicillin cannot be supported by any literature and may be considered idiosyncratic in nature. Despite this, the precise impact of acetate on seizure control may stem from more complex interactions with other metabolites, receptors, or pathways that are yet to be fully elucidated. The undetectable nature of butyrate in the samples may support the claim that the bulk of it is utilized at the site of synthesis in the colon [56]. But this might also hint at specific roles it plays locally in the gut's ecosystem that contribute to overall seizure control.

The enhanced concentration of glutamate and glutamine in the serum of mice pre-treated with VMAN could be attributed to several contributing factors. Firstly, alterations in gut microbiota composition due to VMAN treatment may have indirectly affected the metabolism and availability of neurotransmitter precursors, such as glutamine, which might have led to the observed elevation in circulating concentration. Secondly, the disrupted microbial balance might have influenced the production of metabolites that impact neurotransmitter synthesis, thereby affecting glutamate and glutamine levels in the serum. Additionally, VMAN-induced changes in the gut-brain axis communication could have influenced the release and uptake of glutamate in the brain, subsequently impacting its serum levels. Lastly, the interplay between VMAN-modulated gut microbiota and host immune responses might have indirectly influenced glutamate and glutamine dynamics, as immune factors can modulate neurotransmitter metabolism [7]. These multifaceted mechanisms may have collectively contributed to the observed increase in glutamate and glutamine concentrations, potentially influencing the reversal of the anticonvulsant action of BO and DIA through intricate interactions within the gut-brain axis. This observation is significant due to the fact that glutamate is the main excitatory neurotransmitter, while glutamine is required for its synthesis [16, 21]. 

## Conclusion

From the data obtained from this study, the main constituents of BO are linalool and estragole. BO has anticonvulsant properties. The cocktail of VMAN can significantly deplete the microbiota without causing morbidity and mortality. Administration of cocktail of VMAN and the individual antibiotics does not alter the architecture of the colon. Depleted and altered microbiota significantly reversed the anticonvulsant actions of BO. The concentrations of SCFAs in stool was higher than in serum. Also, the significant concentrations of the presence of the excitatory neurotransmitter; glutamate in the VMAN and vancomycin treated metabolomics may have aided the reversal of the anticonvulsant actions of BO. Administration of BO may not affect the distribution of microorganisms in the microbiota. The increased Firmicutes/Bacteroidetes ratio due to depletion of the microbiota may have also enhanced the reversal of anticonvulsant actions of BO and the standard drug DIA.

### Strengths and limitations of study

The strengths of this study lie in its comprehensive approach to investigating the role of the gut microbiota in the anticonvulsant effect of basil oil in a PTZ induced seizure model. By depleting or altering the microbiota using both cocktail antibiotics and individual antibiotics, the study addresses the complex relationship between gut bacteria and the anticonvulsant properties of BO. The utilization of quantitative reverse transcription-polymerase chain reaction (RT-PCR) to analyse the 16S rRNA gene sequences provides a reliable method to assess the phylogenetic composition of the gut microbiota. Additionally, the incorporation of 1H NMR spectroscopy to determine metabolic profiles adds depth to the understanding of the systemic changes induced by BO treatment.

Despite this, the study has some limitations. First, the use of animal models, while providing valuable insights, may not fully reflect human responses. Secondly, the study focuses on a specific type of seizure model (PTZ-induced seizures), which may not encompass the diversity of seizure types in epilepsy. The study design does not consider potential confounding factors like diet, stress, or other environmental influences on the gut microbiota. Additionally, the mechanisms underlying the observed effects are not fully elucidated, leaving room for speculation. The generalizability of the findings to different types of epilepsy or patient populations needs further exploration.

Notwithstanding these limitations, the study contributes to the growing body of research on the gut-brain axis and its implications for neurological disorders like epilepsy.

## Data Availability

The datasets used and/or analysed during the current study are available from the corresponding author on reasonable request.

## Abbreviations

ASMs: Antiseizure medications
ALT: Alanine aminotransferase
ALP: Alkaline phosphatase
AMPHO B: Amphotericin B
AMP: Ampicillin
ANOVA: Analysis of variance
AST: Aspartate aminotransferase
GBA: Gut-Brain-Axis
BO: Basil oil
Ca^2+^: Calcium ion
CNS: Central nervous system
DIA: Diazepam
DMSO: Dimethyl sulfoxide
DNA: Deoxyribonucleic acids
ENS: Enteric nervous system
GABA: Gamma aminobutyric acid
g-DNA: Genomic deoxyribonucleic acid
GC–MS: Gas chromatography- Mass spectrometry
GGT: Gamma-glutamyl transpeptidase
GIT: Gastrointestinal tract
1H NMR: Proton Nuclear Magnetic Resonance
5HT: 5- Hydroxytryptamine
H&E: Haematoxylin and Eosin
ICR: Institute of Cancer Research
KNUST: Kwame Nkrumah University of Science and Technology
MES: Maximal electrical shock
MET: Metronidazole
Mg/kg: Milligram per kilogram
Na^+^: Sodium ion
NEO: Neomycin
NMR: Nuclear Magnetic Resonance
OECD: Organisation for Economic Cooperation and Development
PBS: Phosphate buffer saline
PCA: Principal Component analysis
PCR: Polymerase chain reaction
p.o: Per os
PTZ: Pentylenetetrazole
RBC: Red blood cell
16S rRNA: 16S ribosomal ribonucleic acid
SCFAs: Short chain fatty acids
SEM: Standard error of mean
USA: United States of America
VAN: Vancomycin
VMAN: Vancomycin, metronidazole, ampicillin, neomycin
